# Tackling Shift Work: Cardiovascular Health in the Auto Industry

**DOI:** 10.3390/healthcare12111097

**Published:** 2024-05-27

**Authors:** Marius Gabriel Bunescu, Veronica Gheorman, Iulia Rahela Marcu, Cristian Virgil Lungulescu, Venera Cristina Dinescu

**Affiliations:** 1 Occupational Medicine Department, Faculty of Medicine, University of Medicine and Pharmacy of Craiova, Petru Rares 2 Street, 200349 Craiova, Romania; marius.bunescu@umfcv.ro; 2 Department 3 Medical Semiology, University of Medicine and Pharmacy of Craiova, Petru Rares 2 Street, 200349 Craiova, Romania; 3 Department of Physical and Rehabilitation Medicine, University of Medicine and Pharmacy of Craiova, Petru Rares 2 Street, 200349 Craiova, Romania; iulia.marcu@umfcv.ro; 4 Oncology Department, University of Medicine and Pharmacy of Craiova, Petru Rares 2 Street, 200349 Craiova, Romania; cristian.lungulescu@umfcv.ro; 5 Department of Health Promotion and Occupational Medicine, University of Medicine and Pharmacy of Craiova, Petru Rares 2 Street, 200349 Craiova, Romania; venera.dinescu@umfcv.ro

**Keywords:** shift work, cardiovascular health, public health

## Abstract

Shift work, particularly in the auto industry, presents significant health challenges, notably in how it impacts cardiovascular health due to irregular work schedules and associated sleep disruptions. This prospective study evaluated 4683 workers from a single Romanian automotive enterprise to investigate the relationship between fixed shift work schedules and cardiovascular health outcomes. Our analysis focused on fixed-shift workers, excluding those on rotating shifts to reduce variability and enhance the clarity of the findings. The findings reveal that night shift workers are at a heightened risk of cardiovascular diseases (CVDs) compared to their day shift counterparts. Night shift workers demonstrated a higher CVD incidence (4.3%) compared to day shift workers (2.6%), with an odds ratio (OR) of 1.68 (95% CI: 1.08 to 2.62, *p* = 0.021). This association remained significant after adjusting for potential confounders, with an adjusted OR of 1.74 (95% CI: 1.09 to 2.75, *p* = 0.019). Male night shift workers exhibited a significantly higher CVD incidence (4.5%) compared to male day shift workers (3.0%), with an OR of 1.75 (95% CI: 1.07 to 2.89, *p* = 0.026). Female night shift workers also showed a higher CVD incidence (3.4%) compared to female day shift workers (1.3%), although this was not statistically significant. These findings underscore the urgent need for targeted interventions and effective strategies to mitigate these risks and promote the cardiovascular health and overall well-being of shift workers in the auto industry. This research contributes to a deeper understanding of how non-traditional work schedules affect health and provides a basis for implementing protective measures in occupational settings.

## 1. Introduction

Shift work in the auto industry presents unique challenges that significantly impact the cardiovascular health and overall well-being of its employees [1]. Characterized by irregular schedules, including night shifts, rotating shifts, and extended hours, such work disrupts the traditional work–life balance, undermining the ability of employees to maintain consistent routines and engage in regular physical activity [2,3,4]. This disruption leads not only to compromised sleep quality but also contributes significantly to increased fatigue and reduced mental alertness, which are critical factors affecting overall health.

Recent studies, including research conducted by AL Travill et al., have documented a higher prevalence of hypertension and other cardiovascular-related conditions among workers exposed to these irregular shift patterns, compared to those adhering to more regular schedules [1]. This finding underscores the profound impact that non-standard work hours have on health, elevating the risk of long-term cardiovascular diseases.

In addition to sleep disruption, shift work in the auto industry poses significant obstacles to maintaining healthy lifestyle choices. Workers find it challenging to access nutritious food options and engage in regular physical activities, factors that are exacerbated by the demanding nature of their work schedules. These lifestyle challenges contribute to broader health implications, including increased risk factors for metabolic disorders and cardiovascular diseases [5,6].

The psychological strain of adapting to non-standard work hours, along with the social isolation often experienced due to conflicting schedules with social and family life, further impacts the emotional well-being of employees [7]. Such stress, when compounded over time, contributes to the overall burden of disease, including chronic conditions like hypertension, coronary artery disease, and obesity [7,8].

Shift work in the auto industry presents distinct challenges that significantly impact employees’ cardiovascular health and overall well-being. Notably, research conducted by Bøggild and Knutsson has demonstrated that shift workers, in general, face a 40% increase in the risk of cardiovascular disease compared to non-shift workers [9].

Specific research within the auto industry, such as the study by Johan Ohlander et al., reveals that various shift work types, especially those excluding nighttime work, are closely linked to hypertension. This association is primarily driven by behavioral mechanisms, including an increased body mass index (BMI), physical inactivity, and disrupted sleep patterns [10]. Furthermore, a systematic review and meta-analysis by Manav V. Vyas et al. has identified a significant association between shift work and major vascular events like myocardial infarction and ischemic stroke, highlighting substantial implications for public policy and occupational health [11].

Adding to the physical stressors are the environmental conditions within auto-manufacturing settings, such as exposure to industrial noise and harmful substances like chemicals and metals, which exacerbate health risks when combined with irregular shift schedules. Moreover, the cyclical nature of the auto industry, coupled with its specific regulatory and safety standards, requires that any interventions be customized to address the unique demands of this sector.

Given these complexities, there is an urgent need for targeted interventions designed to mitigate these risks and enhance the cardiovascular health and general well-being of shift workers. Strategies such as implementing flexible scheduling that allows workers more control over their work hours, developing comprehensive health education programs, and providing robust mental health support are essential. These measures not only address the immediate health concerns but also contribute to the long-term sustainability of the workforce in this demanding industry.

The necessity for immediate and targeted health interventions arises from a growing body of scientific evidence demonstrating the severe and cumulative effects of shift work on cardiovascular health. These health issues carry significant economic implications due to decreased productivity and increased healthcare costs, highlighting the compelling financial incentive to proactively improve worker health outcomes.

Moreover, there is an increasing societal expectation that industries take responsibility for the health and well-being of their employees. The auto industry, with its high-intensity labor and critical operational demands, is particularly positioned to lead by example in this area. By implementing flexible scheduling, health education programs, and mental health support, the industry can mitigate the adverse effects of shift work, leading to a healthier, more productive, and more satisfied workforce.

### 1.1. Purpose and Significance

This study is primarily designed to examine the impact of shift work on cardiovascular health within the auto industry. Our objective is to identify how non-standard and irregular work schedules influence the prevalence of cardiovascular conditions among shift workers, with a particular focus on the direct effects of such schedules on health outcomes like hypertension, coronary disease, and overall cardiovascular risk.

By understanding the specific health impacts linked to shift work, including the roles of disrupted sleep, increased stress and lifestyle challenges, this study aims to offer a robust foundation for developing targeted strategies aimed at enhancing the well-being of shift workers.

### 1.2. Current State of Research

Previous studies have extensively documented the detrimental effects of shift work on sleep quality, mental health, and overall well-being [12,13]. Despite this broad understanding, research specifically focusing on the auto industry is somewhat limited. Moreover, controversies exist regarding the most effective interventions to address the health risks associated with shift work, highlighting the need for further investigation [14,15].

Recent studies have begun to fill these gaps, providing focused insights into the sleep quality among shift workers in specific sectors like the auto industry. For instance, a cross-sectional study conducted by Sagetha J., Preeti P., and Rex Vijay V. assessed sleep quality among workers in the automobile industry in Kanchipuram, Tamil Nadu. Their findings indicated that a significant portion of shift workers, particularly younger employees aged 18–25, experienced poor sleep quality, which correlated with physical health issues such as frequent nocturnal awakenings and systemic illnesses [16]. This study underscores the impact of shift work on sleep disruption and its broader implications for workplace efficiency and safety.

Similarly, research by Kawada T., Shimizu T., Kuratomi Y., et al. explored the effects of different shift schedules on sleep duration among auto industry workers. Utilizing accelerometers to measure sleep, their study revealed that sleep durations varied minimally across different shifts, although workers tended to sleep less during night shifts compared to morning and day shifts. This suggests that while rotating shifts do not drastically change total sleep time, the timing of shifts can significantly affect sleep quality [17].

These studies emphasize the need for strategic intervention planning that focuses on mitigating the physical and psychological stressors associated with shift work. Protective efforts such as optimizing shift scheduling, educating workers on healthy sleep practices, and monitoring sleep patterns should be considered critical components of workplace health initiatives. By incorporating such data-driven strategies, employers in the auto industry can better support the well-being of their workforce, potentially reducing the incidence of sleep-related impairments and their consequent impact on productivity and health.

In light of these findings, it is clear that while general research on the impact of shift work on health is extensive, targeted studies within the auto industry are crucial for developing effective interventions tailored to the unique challenges faced by this sector. As such, there remains an urgent need to continue exploring and addressing the specific health risks associated with various shift work patterns in the automotive industry.

## 2. Materials and Methods

### 2.1. Study Setting and Enterprise Selection

The study was conducted at a Romanian automotive manufacturing enterprise located in Dolj county, chosen for several strategic reasons that are crucial for achieving the research objectives. This section details the rationale behind selecting this particular enterprise, ensuring transparency in our study design and the broader applicability of our findings.

Access and Cooperation: The chosen enterprise provided unparalleled access to comprehensive health and employment records, essential for this study. Their proactive cooperation and established frameworks for academic collaboration facilitated extensive data collection, crucial for the robust analysis of shift work’s impact on cardiovascular health.

Workforce Size and Diversity: The enterprise boasts a workforce of over 4600 employees, making it one of the largest in the Dolj region’s automotive industry. The significant size and demographic diversity of the workforce provide sufficient statistical power to detect meaningful health effects, ensuring the results are statistically robust and reflective of varied employee experiences.

Industry Representation: Representing large-scale automotive manufacturing operations typical of Eastern Europe, this enterprise features standard manufacturing processes, work shift patterns, and employee demographics common across the industry. This representativeness strengthens the generalizability of our findings, allowing them to be applicable to other enterprises within the same industrial sector, potentially guiding industry-wide health and safety practices.

Geographical and Economic Context: Romania’s burgeoning automotive industry and evolving occupational health regulations present a unique context for this study. The geographic and economic factors make our findings relevant not only to this specific enterprise but also to the broader industry in Eastern Europe and similar regions. This relevance is critical for informing policy and practical applications in occupational health within the automotive sector.

### 2.2. Data Collection

To achieve our objectives, we conducted a prospective evaluation of 4683 workers in the automotive industry aged over 18 and under 65 from a single Romanian enterprise between December 2023 and March 2024. The inclusion criteria comprised individuals who were at least 18 years old, willing to participate in the study, and who provided written informed consent. Each participant provided written informed consent, and the study received approval from the Ethics Committee of the University of Medicine and Pharmacy of Craiova (No. 146/28 November 2023).

Data regarding cardiovascular health conditions such as arterial hypertension, rhythm disorders, and heart failure were collected through clinical assessments conducted at the company’s health center. Each assessment included blood pressure measurements, ECG tests, and a review of medical histories confirmed by healthcare professionals.

Data from clinical assessments were recorded electronically in a secure, anonymized database to maintain participant confidentiality. The information was then verified and validated by medical experts to ensure accuracy before analysis. This process included cross-checking the medical records with the data entered into the system and resolving any discrepancies through follow-up with the health center staff.

In addition to cardiovascular assessments, we also monitored other relevant health metrics that could influence cardiovascular outcomes, including body mass index (BMI), lipid profiles, and glucose levels. These metrics were assessed using standardized equipment and procedures to ensure consistency across all evaluations.

### 2.3. Study Population and Design

This study specifically focused on fixed-shift workers within the automotive industry to assess the impact of consistent shift schedules on cardiovascular health. It is important to note that rotating shift workers were not included in our analysis. This decision was strategic, aiming at minimizing variability in work patterns and enhancing our ability to isolate and examine the effects of non-rotating (fixed) shift schedules. This exclusion criterion has been clearly specified to ensure the clarity of our study’s scope and the interpretability of its findings.

### 2.4. Shift Work Definitions and Schedules

In our study, “day shift” is defined as a work period from 7 a.m. to 3 p.m., and “night shift” is defined as a work period from 11 p.m. to 7 a.m. To minimize variability and better isolate the impact of shift work on cardiovascular health, our study design specifically addresses the regularity and consistency of these shifts.

### 2.5. Definition of Seniority in Work

In this study, ‘seniority in work’ is specifically defined as the duration of time an individual has been employed in their current role at the enterprise under investigation. This definition does not consider previous employment rolls or positions outside the current organization. It is intended to focus on the current job conditions and their impact on the health of the employees.

The rationale behind this definition is to minimize confounding variables that might arise from varied past employment experiences and to more accurately assess the direct impact of current job roles and work conditions on cardiovascular health. This specific focus allows us to isolate the influence of the current work environment and practices at the enterprise on employee health outcomes.

### 2.6. Statistical Analysis

A frequency analysis was conducted to assess variables such as sex, age, seniority in work, type of work, and work schedule. We investigated the prevalence of key cardiovascular diseases, including arterial hypertension, rhythm disorders, heart failure, and acute myocardial infarction, as well as diabetes, dyslipidemia, and obesity, as risk factors for cardiovascular disease. Cardiovascular conditions were identified based on diagnostic codes from health records and verified by consulting the participants’ primary healthcare providers.

The risk analysis was carried out by calculating the odds ratio (OR) and 95% confidence intervals (95% CIs), with the statistical significance set at *p* < 0.05. We compared the frequency of CVD between night shift workers and day workers (one shift) and calculated both a crude and age-adjusted OR.

The analysis was performed using MedCalc version 22.0, with a focus on enhancing the understanding of CVD prevalence and risk factors among automotive industry workers subjected to varying work schedules, particularly night shifts.

## 3. Results

The results of the study provide a detailed overview of the demographic distribution, prevalence of cardiovascular disease (CVD), and associated risk factors among workers in the auto industry (Table 1). This section presents the findings derived from the analysis of the collected data, offering insights into the cardiovascular health landscape within this specific occupational setting.

### 3.1. Demographic Distribution and Occupational Characteristics

Gender Distribution and Occupational Roles

The workforce predominantly comprises male employees, constituting 79.3% (3713 workers) of the total workforce. Female representation stands at 20.7% (970 workers). Interestingly, the majority of both male and female workers are engaged in physically demanding roles, with 88.10% (4128 workers) in such positions. Sedentary roles are less common but still significant, comprising 11.90% (555 workers) of the workforce.

### 3.2. Age Distribution and Shift Work Patterns

The average age of the workforce is 37.78 ± 9.17 years, with the majority falling below 50 years (89.10%, 4173 workers). Workers aged between 50–65 years constitute 10.90% (510 workers). Night shift work is predominant, with 81.20% (3802 workers) of the workforce engaged in night shifts, while day shift workers represent 18.80% (881 workers).

### 3.3. Cardiovascular Disease (CVD) Prevalence

The prevalence of CVD among the workforce is 4.00% (187 workers). Hypertension is the most prevalent CVD, affecting 3.90% (181 workers) of the workforce. Rhythm disorders, atrial fibrillation, heart failure, and myocardial infarction are less common but still present.

### 3.4. Cardiovascular Risk Factors

The workforce exhibits several cardiovascular risk factors, including diabetes (3.8%, 177 workers), obesity (4.6%, 217 workers), and dyslipidemia (0.02%, 10 workers).

To estimate the risk, odds ratios (ORs) and 95% confidence intervals (CIs) for ORs were used, with the significance set at *p* < 0.05. For comparing averages, a *t*-test was utilized, and the results showed differences, accompanied by 95% confidence intervals (CIs) for these differences and *p*-values, significant at *p* < 0.05

Table 2 delineates the incidence of cardiovascular disease (CVD) among workers in the auto industry, providing a nuanced perspective across multiple parameters. This analysis aims to uncover patterns and correlations that shed light on the cardiovascular health landscape within this occupational sector.

1. Overall CVD Incidence

Total CVD Cases: A total of 187 cases of CVD were identified, constituting 4% of the workforce.

Without CVD: The majority, 4496 workers (96%), were found to be free from CVD.

While the overall incidence of CVD is relatively low at 4%, it is crucial to recognize that even this moderate prevalence signifies a substantial number of workers affected. The large majority without CVD provides a benchmark against which to compare and assess risk factors.

2. Gender and CVD

Male Workers: A higher incidence of CVD was observed among male workers, accounting for 2.9% of cases (159 workers).

The gender disparity in CVD incidence suggests potential gender-specific risk factors or vulnerabilities that warrant further investigation. The statistically significant difference (OR = 1.51, *p* = 0.048) underscores the need for targeted gender-specific interventions.

3. Age and CVD

<50 years: A significant proportion of younger workers (<50 years) were affected by CVD, with an incidence of 2.5%.

The elevated risk among younger workers challenges conventional perceptions of CVD as an age-related disease. This finding emphasizes the importance of early detection and intervention strategies tailored to younger age groups.

The data on workers aged 50 years and older show a higher incidence rate of cardiovascular disease (CVD) compared to younger workers. Of the 510 workers in this age group, 83 (or 16.3%) were affected by CVD. This indicates a significant burden of the disease in middle-aged to older adults, which aligns with the conventional understanding that the risk of CVD increases with age.

4. Seniority in Work and CVD

0–10 years: Workers with 0–10 years of seniority exhibited a higher incidence of CVD at 2.3%.

A shorter tenure in the workforce appears to correlate with increased CVD risk. This finding suggests that newer entrants to the industry may face unique challenges or exposures that contribute to cardiovascular health risks.

For workers with more than 10 years of seniority, the data show a notably higher incidence of cardiovascular disease (CVD), at 7.0%. Among the 1718 workers in this category, 120 experienced CVD. This increased rate among those with a longer tenure could suggest several underlying factors.

Firstly, the accumulation of long-term stress and possible exposure to occupational hazards over extended periods could contribute significantly to the elevated risk. Workers with greater seniority might also be older, which naturally aligns with an increased likelihood of developing CVD due to age-related physiological changes.

Additionally, this pattern might reflect lifestyle factors that can worsen over time, such as sedentary work habits, prolonged exposure to workplace stress, or neglect of personal health due to demanding work commitments. This indicates a critical need for ongoing health and wellness programs that address the specific needs of long-term employees.

Understanding the increased risk in this group also highlights the importance of implementing preventive measures early in an employee’s career and maintaining these interventions as they gain tenure. This approach could help mitigate the higher observed incidence of CVD in workers with more than 10 years of seniority, promoting healthier, longer careers.

5. Type of Work and CVD

Physically Demanding Roles: Workers engaged in physically demanding roles had a CVD incidence of 4.3%. This contrasts sharply with those in sedentary roles, where the incidence was only 1.8%. Emphasizing the disparity in these rates can help to underline the different health challenges faced by workers in varying occupational settings.

The heightened risk associated with physically demanding roles underscores the potential cardiovascular strain induced by strenuous work. This observation calls for ergonomic assessments and interventions to mitigate physical stressors.

6. Working Program and CVD

Night Shift: Night shift workers demonstrated a higher CVD incidence at 4.3% compared to day shift workers at 2.6%.

The association between night shift work and increased CVD risk suggests that circadian rhythm disruptions may play a role in cardiovascular health. This finding highlights the need for scheduling considerations and lifestyle modifications for night shift workers.

Table 3 compares the incidence of cardiovascular disease (CVD) between night and day shift workers across various parameters, including gender, age, seniority in work, and type of work. This comparative analysis aims to elucidate potential differences in CVD risk profiles between the two shift groups.

1. Gender and Shift Work

Male Workers: Night shift male workers exhibited a higher CVD incidence (4.5%) compared to day shift male workers (3.0%).

The elevated risk among male night shift workers suggests that night shift work may exacerbate cardiovascular risk factors more significantly among male workers than their day shift counterparts (OR = 1.52, *p* = 0.046).

Female Workers: Night shift female workers demonstrated a higher CVD incidence (3.4%) compared to day shift female workers (1.3%), although this difference was not statistically significant.

The smaller sample size of female workers may contribute to the lack of statistical significance, warranting further investigation into gender-specific risks associated with shift work.

2. Age and Shift Work

<50 years: Night shift workers below 50 years showed a higher CVD incidence (2.7%) compared to day shift workers (1.6%), but the difference was not statistically significant.

The non-significant difference suggests that younger workers may be equally vulnerable to CVD risks regardless of shift work, highlighting the need for age-specific interventions.

50–65 years: Night shift workers aged 50–65 years exhibited a higher CVD incidence (17.1%) compared to their day shift counterparts (12.0%), although this was not statistically significant.

The elevated risk among older night shift workers underscores the potential compounding effect of age and night shift work on CVD risk, warranting age-specific interventions.

3. Seniority in Work and Shift Work

0–10 years: Night shift workers with 0–10 years of seniority had a significantly higher CVD incidence (2.5%) compared to day shift workers (0.8%).

The significant difference suggests that newer night shift workers may face unique challenges or exposures that increase CVD risk (OR = 3.16, *p* = 0.026).

>10 years: Night shift workers with more than 10 years of seniority exhibited a higher CVD incidence (7.6%) compared to day shift workers (4.8%), with a statistically significant difference.

The increased risk among longer-tenured night shift workers highlights the potential long-term impact of night shift work on cardiovascular health (OR = 1.62, *p* = 0.042).

4. Type of Work and Shift Work

Physically Demanding Roles: Both night and day shift workers in physically demanding roles showed similar CVD incidences (4.3% and 4.2%, respectively).

The comparable risk profiles between shift groups suggest that the nature of the work may be a more significant factor than the shift pattern itself in these roles.

Sedentary Roles: Night shift workers in sedentary roles exhibited a significantly higher CVD incidence (6.7%) compared to day shift workers (1.5%).

The pronounced difference suggests that sedentary night shift work may pose unique cardiovascular health risks (OR = 4.62, *p* = 0.04).

The logistic regression analysis evaluates the association between night shift work and cardiovascular disease (CVD) incidence, providing insights into the odds ratios, confidence intervals, regression coefficients, standard errors and *p*-values across different groups (Table 4).

1. Overall Night Shift and CVD Incidence

All Workers (not age adjusted): The odds ratio (OR) of developing CVD for all night shift workers is 1.68 (95% CI: 1.08 to 2.62), with a regression coefficient of 0.52.

Night shift work is associated with 1.68 times higher odds of developing CVD, after controlling for other factors (*p* = 0.021).

All Workers: Adjusted for all variables, the OR increases slightly to 1.74 (95% CI: 1.09 to 2.75).

Even after adjusting for potential confounders, the association between night shift work and CVD remains significant (*p* = 0.019).

2. Gender-Specific Night Shift and CVD Incidence

Male Workers: The OR for male night shift workers is 1.75 (95% CI: 1.07 to 2.89).

Male night shift workers have 1.75 times higher odds of developing CVD compared to male day shift workers, after adjusting for other variables (*p* = 0.026).

Female Workers: The OR for female night shift workers is 1.59 (95% CI: 0.45 to 5.61), although not statistically significant.

Legend—* = not age adjusted; blue square = OR; blue bars = CI95% for OR; vertical red line at OR = 1 meaning limit of risk significance for OR, OR > 1, and CI95% > 1 was considered significant, with *p* < 0.05.

Overall, Night Shift, and CVD Risk: Night shift work is independently associated with an increased risk of CVD among all workers, with an OR ranging from 1.68 to 1.74 across different models. This finding underscores the importance of considering shift work patterns as a potential risk factor in CVD prevention strategies.

Gender-Specific Night Shift and CVD Risk: Male night shift workers exhibit a significantly higher risk of CVD compared to their day shift counterparts, with an OR of 1.75. In contrast, the association is not statistically significant among female workers, potentially due to the smaller sample size.

Adjustment for Other Variables: The consistency of the ORs across different models suggests that the association between night shift work and CVD risk is robust, even after adjusting for potential confounders.

**Figure 1:**
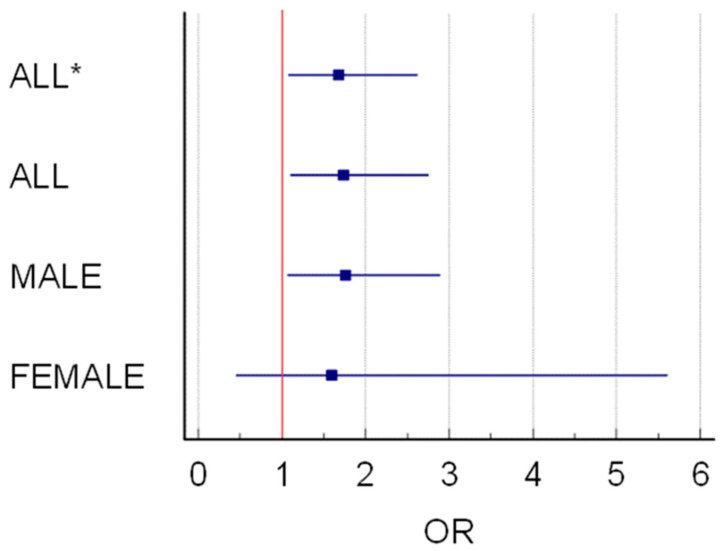
Age-adjusted risk of CVD for night shift workers.

The logistic regression analysis reaffirms the association between night shift work and increased CVD risk, particularly among male workers (Figure 1). While the exact mechanisms underlying this association require further investigation, the findings underscore the importance of targeted interventions and regular cardiovascular health screenings for night shift workers in the auto industry.

## 4. Discussion

Our study aimed to investigate the demographic distribution, occupational characteristics, and cardiovascular health of workers in the auto industry, with a particular focus on the impact of shift work on cardiovascular disease (CVD) prevalence. The findings reveal several important insights and implications for occupational health and safety.

### 4.1. Gender Distribution and Occupational Roles

The workforce is predominantly male (79.3%), and both male and female workers are largely engaged in physically demanding roles (88.10%). This gender disparity aligns with previous research indicating male dominance in physically intensive industrial sectors [18]. The higher incidence of CVD among male workers (2.9%) compared to female workers highlights the need for gender-specific health interventions, considering men’s higher exposure to physical strain and possibly different lifestyle factors [19].

### 4.2. Age Distribution and Shift Work Patterns

The average age of the workforce is 37.78 ± 9.17 years, with a majority (89.10%) under 50 years old. Despite the relatively young workforce, a significant proportion of younger workers (<50 years) are affected by CVD (2.5%). This contradicts the conventional view that CVD primarily affects older individuals and underscores the necessity for early intervention strategies [20]. The elevated CVD prevalence in older workers (16.3% in those aged 50–65 years) corroborates established findings on age-related CVD risk [21].

### 4.3. Shift Work and Cardiovascular Health

Night shift work is predominant, involving 81.20% of the workforce, and is associated with a higher CVD incidence (4.3%) compared to day shifts (2.6%). The increased CVD risk among night shift workers, particularly males, suggests that circadian rhythm disruptions contribute significantly to cardiovascular strain [22]. This finding is consistent with evidence that shift work, especially night shifts, is linked to adverse cardiovascular outcomes [23].

### 4.4. Cardiovascular Disease Prevalence and Risk Factors

The overall prevalence of CVD in the workforce is 4%, with hypertension being the most common condition (3.9%). The presence of risk factors such as diabetes (3.8%), obesity (4.6%), and dyslipidemia (0.02%) align with broader epidemiological trends and highlights the need for comprehensive workplace health programs [24].

### 4.5. Occupational Role and CVD Incidence

Workers in physically demanding roles exhibit a higher CVD incidence (4.3%) compared to those in sedentary roles (1.8%). This suggests that physical strain and related stressors may elevate cardiovascular risk [25]. Conversely, the lower CVD prevalence in sedentary workers may reflect reduced physical exertion, though prolonged inactivity has its own health risks [26].

### 4.6. Seniority in Work and CVD

The incidence of CVD is notably higher among workers with more than 10 years of seniority (7.0%) compared to those with less than 10 years (2.3%). This trend indicates that prolonged exposure to occupational hazards and stressors over time may increase cardiovascular risk [27]. Long-term employees may benefit from targeted interventions to mitigate these risks, including stress management and ergonomic adjustments.

### 4.7. Potential Long-Term Implications:

Chronic Health Conditions: The cumulative effects of prolonged non-traditional shift work can contribute to the development of chronic health conditions, including persistent hypertension, cardiovascular diseases, and metabolic disorders, impacting long-term cardiovascular health [28].

Reduced Life Expectancy: Studies have suggested that individuals engaged in non-traditional shift work may have a reduced life expectancy attributed to the increased risk of cardiovascular events and metabolic complications [29,30].

Impact on Quality of Life: The psychological and cardiovascular implications of non-traditional shift work can significantly impact an individual’s quality of life, potentially leading to decreased mobility, energy levels, and overall well-being over time [31].

### 4.8. Addressing Cardiovascular Health

Shift work has been associated with an increased risk of cardiovascular issues, and several potential mechanisms may contribute to this relationship. Additionally, it is crucial to understand the importance of cardiovascular health in the context of shift work and the implications for the overall well-being of shift workers. Here is an investigation into the potential mechanisms behind the association between shift work and cardiovascular issues, as well as the significance of cardiovascular health in the context of non-traditional work schedules:

#### Potential Mechanisms:

1. Disruption of Circadian Rhythms: Shift work often leads to disruptions in circadian rhythms, impacting the body’s natural sleep–wake cycle [32]. This disruption can affect hormonal regulation, including the release of cortisol and melatonin, which play roles in managing stress and supporting the overall physiological balance.

2. Sleep Deprivation and Quality: Non-traditional work schedules can lead to inadequate or poor-quality sleep, which is a known risk factor for cardiovascular issues [33]. Sleep deprivation and irregular sleep patterns can lead to increased systemic inflammation, insulin resistance, and the dysregulation of metabolic processes, all of which contribute to cardiovascular risk [34,35].

3. Changes in Lifestyle and Health Behaviors: Shift work can result in changes in lifestyle and health behaviors, including disrupted mealtimes, irregular physical activity patterns, and reliance on convenience foods [36]. These factors can contribute to the development of risk factors such as obesity, high blood pressure, and dyslipidemia, all of which are associated with cardiovascular disease.

4. Increased Stress and Psychosocial Factors: Working non-traditional shifts can lead to increased stress and exposure to psychosocial factors that may impact cardiovascular health [37,38]. Chronic stress and social disruption can contribute to elevated blood pressure, increased sympathetic nervous system activity, and unhealthy coping behaviors, all of which can negatively impact cardiovascular function.

5. Reduced Access to Healthcare and Preventive Services: Shift work may limit access to regular healthcare services and preventive screenings due to scheduling conflicts [39]. Reduced access to routine medical care can delay the early detection and management of cardiovascular risk factors, potentially leading to poorer cardiovascular outcomes [40].

### 4.9. Importance of Cardiovascular Health in the Context of Shift Work

The significance of cardiovascular health in the context of shift work cannot be overstated, as the well-being of shift workers is intricately linked to their cardiovascular function [41]:

1. Occupational Safety and Performance: Cardiovascular health is critical for maintaining the safety and performance of shift workers, especially those engaged in physically demanding or high-stress occupations. Good cardiovascular function supports endurance, recovery, and resilience in the face of occupational challenges [29,41].

2. Overall Health and Well-Being: Cardiovascular health is a fundamental component of overall well-being. Maintaining optimal cardiovascular function is essential for minimizing the risk of chronic diseases, supporting longevity, and ensuring that individuals can lead healthy and fulfilling lives both inside and outside the workplace [42].

3. Productivity and Absenteeism: Cardiovascular issues can significantly impact productivity and absenteeism among shift workers [43]. Poor cardiovascular health may lead to an increased likelihood of sick leave, reduced work performance, and higher healthcare costs, all of which can affect both the individual and the organization.

4. Long-Term Health Outcomes: The impact of poor cardiovascular health extends beyond the immediate effects on work performance [44]. Cardiovascular issues can lead to long-term health complications, including heart disease, stroke, and other related conditions, which can have profound implications for the overall quality of life and longevity of shift workers.

Understanding the importance of cardiovascular health in the context of shift work is critical for safeguarding the well-being of shift workers, promoting occupational safety and performance, and supporting long-term health outcomes. Efforts to mitigate the impact of non-traditional work schedules on cardiovascular health should encompass strategies to address sleep disturbances, promote healthy lifestyle behaviors, provide access to preventive healthcare services, and raise awareness about the significant role of cardiovascular health in preserving the overall well-being of shift workers.

### 4.10. Strategic Interventions

While the primary focus of this research does not include the direct evaluation of interventions, based on our findings, we propose several strategies for intervention designed to mitigate the adverse effects identified. These interventions are derived from a combination of our research outcomes and established best practices within occupational health. They are intended as recommendations for future implementation and further study, aiming to address the specific needs identified through our analysis.

1. Promoting Healthy Lifestyle Behaviors:
  - Encourage shift workers to engage in regular physical activity, even if it means adjusting workout schedules to fit their shift patterns. Providing access to on-site fitness facilities or partnerships with local gyms can facilitate this.
  - Educate employees about the importance of maintaining a balanced diet despite working non-standard shifts. Offering healthy food options during overnight shifts and providing nutritional resources can help support healthy eating habits [36].
  - Promote strategies for stress management and relaxation techniques, such as mindfulness training, yoga classes, or access to counseling services to help employees cope with the stress associated with their work schedules [45].
2. Sleep Hygiene and Management:
  - Provide education on good sleep hygiene practices for shift workers, including creating a conducive sleep environment, maintaining consistent sleep schedules, and minimizing disruptions during sleep hours [46].
  - Consider implementing policies that allow for adequate time for sleep between shift rotations and provide guidance on how to optimize sleep quality and duration [46].
3. Flexible Scheduling and Shift Design:
  - Explore opportunities for flexible scheduling, such as compressed workweeks or allowing employees to have a say in their shift preferences where possible. This can help reduce the frequency of rotating shifts, which can be particularly disruptive to circadian rhythms.
  - Consider aligning shift rotations in a forward-rotating manner (morning, evening, night shifts) to allow the body’s internal clock to adjust more effectively, rather than rotating shifts in a reverse order [47].
4. Cardiovascular Risk Assessments and Monitoring:
  - Offer regular cardiovascular risk assessments, including screenings for blood pressure, cholesterol levels, and weight management programs, to help identify and manage potential risk factors early on [48].
  - Provide access to occupational health services that specifically address the unique needs and risk factors faced by shift workers, including cardiovascular risk factors [49].
5. Employee Assistance Programs and Resources:
  - Establish access to employee assistance programs that offer support for mental health, stress management, and coping with the challenges associated with shift work [50].
  - Provide access to resources such as financial planning assistance, legal services, and support for family-related issues to help alleviate potential stressors that can impact cardiovascular health.
6. Worksite Health Promotion Initiatives:
  - Implement worksite health promotion initiatives that focus on cardiovascular health, such as organizing health fairs, offering smoking cessation programs, and providing access to flu shots and other preventive healthcare services.
  - Foster a workplace culture that values and prioritizes employee well-being, providing opportunities for physical activity, healthy eating options, and wellness challenges to engage shift workers in positive health behaviors.
7. Education and Training:
  - Offer educational programs on cardiovascular health, addressing topics such as the impact of shift work on cardiovascular function, the importance of stress management, and strategies for maintaining a healthy lifestyle despite non-traditional work schedules [44].
  - Provide training for managers and supervisors on recognizing signs of fatigue, stress, and potential health issues in shift workers, while also promoting supportive and flexible leadership practices.
8. Access to Healthcare and Preventive Services:
  - Ensure that shift workers have access to healthcare services outside of standard business hours. This might include accommodating alternate appointment times with healthcare providers or offering telemedicine options for remote consultations.
  - Provide information about the availability of community health resources, such as local clinics and support groups, to supplement access to healthcare services.
9. Supportive Organizational Policies:
  - Establish policies that promote a healthy work–life balance, including measures to limit excessive overtime, provide adequate rest periods between shifts, and consider shift preferences when feasible.
  - Recognize the unique challenges faced by shift workers and create a supportive work environment that fosters open communication, active listening, and responsive action to address employee concerns related to their work schedules.

By implementing these recommendations and interventions, organizations can support employees working non-standard shifts in reducing cardiovascular risks, promoting overall health, and creating a workplace environment that prioritizes the well-being of all workers, regardless of their work schedule.

Future Directions:

The continued research and advancements in addressing the cardiovascular health challenges related to shift work hold significant promise for improving the well-being of shift workers in the auto industry and beyond. Here are some potential areas for further research and development:

1. Personalized Interventions: There is a growing need for personalized interventions tailored to the unique needs of individual shift workers. Advancements in technology, such as wearable health-monitoring devices, could enable the collection of personalized data on sleep patterns, stress levels, and cardiovascular health indicators. This data can be utilized to design targeted interventions that address specific challenges faced by each shift worker, leading to more effective health outcomes.

2. Integration of Mental Health Support: Further research into the integration of mental health support within the context of shift work is crucial. This includes investigating the effectiveness of mental health screenings, early intervention programs, and destigmatization efforts within the workplace. Understanding the most effective ways to promote mental well-being, resilience, and coping strategies among shift workers can lead to comprehensive support systems that address both psychological and cardiovascular health challenges.

3. Chronobiology and Shift Work: Advancements in chronobiology research can provide deeper insights into the interactions between shift work schedules and the body’s internal biological clock. Understanding the impact of different shift patterns on circadian rhythms, sleep quality, and cardiovascular health can inform the development of optimized shift schedules that minimize disruptions to the body’s natural rhythms. Additionally, exploring chronotherapy and light exposure interventions tailored to specific shift schedules could lead to innovative approaches for mitigating the adverse effects of shift work.

4. Telehealth and Virtual Support: With the increasing prevalence of telehealth services, further research can explore the effectiveness of virtual mental health support and counseling for shift workers. Investigating the accessibility and efficacy of telehealth interventions for addressing stress, anxiety, and sleep disorders among shift workers can open up new avenues for providing convenient and timely support, regardless of employees’ work schedules.

5. Integrative Wellness Programs: Research into integrative wellness programs that combine physical activity, nutrition, stress management, and mental health support can provide insights into holistic approaches to promoting well-being among shift workers. Understanding the synergistic effects of such multi-component interventions can guide the development of comprehensive wellness programs tailored to the specific needs of shift workers in the auto industry and other sectors.

6. Long-Term Health Outcomes: Further longitudinal studies focusing on the long-term health outcomes of shift workers, including cardiovascular disease risk, mental health disorders, and overall mortality rates, are essential. This research can shed light on the cumulative impacts of shift work on health and well-being, influencing policy decisions, workplace practices, and healthcare initiatives aimed at preventing and managing the long-term effects of working non-traditional schedules.

By advancing research in these areas, the auto industry and other sectors can gain valuable insights into how to effectively address the psychological and cardiovascular health challenges associated with shift work. These insights can inform the development of targeted interventions and evidence-based policies and supportive workplace practices that foster the well-being and resilience of shift workers, ultimately contributing to a healthier and more productive workforce.

Limitations of the study

This study, while providing valuable insights into the impact of occupational factors on cardiovascular disease (CVD) in automotive industry workers, acknowledges certain limitations in the scope of the analyzed variables. Notably, the study did not include a family history of disease or smoking and drinking habits, among other lifestyle factors which are recognized as significant contributors to CVD risk.

Future Research

In recognition of these limitations, we propose that future research efforts in this area should aim to incorporate a broader spectrum of confounding factors. This would enrich the understanding of the complex interplay between occupational stressors and personal lifestyle choices in the development of CVD. Enhancing data collection methods to include detailed personal and familial health histories alongside occupational data will be crucial in achieving a more holistic view of the factors affecting CVD among industrial workers.

## 5. Conclusions

Our study has provided critical insights into the prevalence of cardiovascular diseases (CVDs) and associated risk factors among workers in the auto industry, particularly highlighting the impact of shift work on these outcomes. The findings demonstrate a significant association between night shift work and increased cardiovascular risk, underscoring the need for targeted interventions to mitigate these risks.

We observed that night shift workers exhibited a higher incidence of CVD compared to day shift workers, a discovery that calls attention to the specific challenges posed by nocturnal work schedules. This aligns with the existing literature that links disruptions of circadian rhythms to adverse cardiovascular outcomes. This study also highlighted the prevalence of risk factors such as hypertension, diabetes, and obesity, which are exacerbated by the irregular work hours and lifestyle constraints faced by shift workers.

Based on these findings, we advocate for the implementation of comprehensive health management strategies within the auto industry. These should include regular cardiovascular health screenings, enhanced access to healthy lifestyle options in the workplace, and shift scheduling that minimizes circadian disruption. Furthermore, the study points to the need for policies that support a healthier work environment, such as flexible work arrangements and better health education tailored to the needs of shift workers.

Looking forward, further research should explore the longitudinal effects of shift work on cardiovascular health and examine the efficacy of specific health interventions. This could include randomized controlled trials of lifestyle modification programs or ergonomic improvements in the workplace. Additionally, extending the study to include a wider range of confounding factors, such as genetic predispositions and broader lifestyle habits, would provide a more comprehensive understanding of the risks associated with shift work.

In conclusion, our research contributes to the growing body of evidence on the occupational health challenges faced by auto industry workers, emphasizing the critical need for industry-specific health promotion and disease prevention strategies. By addressing the unique conditions of shift work, stakeholders can better protect and enhance the health and well-being of their employees.

## Figures and Tables

**Table 1:** Key characteristics of shift work in auto industry.

Parameters	N	%	Parameters	N	%
Gender			Type of work		
Male	3713	79.3	Physical	4128	88.1
Female	970	20.7	Sedentary	555	11.9
Age			Working program		
Average (years ± SD)	37.78 ± 9.17		Night shift	3802	81.2
Age < 50 years	4173	89.1	Day shift	881	18.8
Age 50–65 years	510	10.9			
Group age			Cardiovascular disease (CVD)		
18–19	58	1.2	All CVDs	187	4
20–29	882	18.8	Hypertension	181	3.9
30–39	1748	37.3	Rhythm disorders	24	0.5
40–49	1485	31.7	Atrial fibrillation	11	0.2
50–59	495	10.6	Heart failure	25	0.5
60–65	15	0.3	Myocardial infarction	9	0.2
Seniority in work			Risk Factors		
Average (years ± SD)	7.82 ± 5.69		Diabetes	177	3.8
<0–10 years	2964	63.3	Dyslipidemia	10	0.02
>10 years	1718	36.7	Obesity	217	4.6

(SD—standard deviation).

**Table 2:** Incidence of cardiovascular disease (CVD) among auto industry workers.

Parametrs	CVD	No CVD	Total	Risk		
	N (%)	N (%)	N	OR /Differences	IC95%	*p*
All	187 (4.0)	4496 (96.0)	4683			
Gender						
Male *	159 (2.9)	3554 (97.1)	3713	1.51	1.01–2.26	0.048
Female	28 (4.3)	942 (95.7)	970			
Age						
Average age	48.18 ± 6.96	37.35 ± 8.99		−10.84 y	−12.27–9.41	<0.001
<50 years	104 (2.5)	4069 (97.5)	4173	7.61	5.65–10.42	<0.001
50–65 * years	83 (16.3)	427 (83.7)	510			
Seniority in work						
Years	11.37 ± 5.07	7.67 ± 5.67		−3.7 y	−4.52–(−2.87)	<0.001
0–10 years	67 (2.3)	2897 (97.7)	2964	3.25	2.39–4.41	<0.001
>10 years *	120 (7.0)	1598 (93.0)	1718			
Type of work						
Physically *	177 (4.3)	3951 (95.7)	4128	2.44	1.28–4.65	0.007
Sedentary	10 (1.8)	545 (98.2)	555			
Working program						
Night shif t *	164 (4.3)	3638 (95.7)	3802	1.682	1.08–2.62	0.021
Day shift	23 (2.6)	858 (97.4)	881			

* passive work (IT, administrative), SD—standard deviation.

**Table 3:** Comparison of CVD incidence between night and day shift workers.

Parameters	Night Shift with CVD	Day Shift with CVD	Total	Risk		
	N %	N %	N	OR /Differences	IC95%	*p*
Gender						
Male	139 4.5	20 3.0	159	1.52	1.04–2.44	0.046
Female	25 3.4%	3 1.3%	28	2.55	0.76–8.54	0.127
Age						
Average age (years ± SD)	37.76 ± 9.29	37.86 ± 8.62		0.1	−0.57–0.77	0.77
<50 years	91 2.7%	13 1.6%	104	1.67	0.93–3.01	0.085
50–65 years	73 17.1%	10 12.0%	83	1.51	0.74–3.05	0.257
Seniority in work						
Years	7.59 ± 5.57	8.8 ± 6.1		1.21 y	0.79–1.63	<0.001
0–10 years	63 2.5%	4 0.8%	67	3.16	1.14–8.72	0.026
>10 years	101 7.6%	19 4.8%	120	1.62	1.05–2.53	0.042
Type of work						
Physical	162 4.3%	15 4.2%	177	1.02	0.59–1.75	0.94
Sedentary	8 6.7%	2 1.5%	10	4.62	1.03–20.76	0.04

**Table 4:** Logistic regression analysis of CVD incidence and night shift work.

	Odds Ratio	95% CI	Regression Coefficients	Std. Error	*p*
Night Shift					
ALL *	1.68	1.08 to 2.62	0.52	0.23	0.021
ALL	1.74	1.09–2.75	0.55	0.24	0.019
MALE	1.75	1.07 to 2.89	0.56	0.25	0.026
FEMALE	1.59	0.45 to 5.61	0.46	0.64	0.47

* Not age adjusted.

## Data Availability

The data presented in this study are available on request from the corresponding author.
